# Socioeconomic determinants of global distribution of multiple sclerosis: an ecological investigation based on Global Burden of Disease data

**DOI:** 10.1186/s12883-021-02170-3

**Published:** 2021-04-01

**Authors:** Vahid Kazemi Moghaddam, Aisha S. Dickerson, Edris Bazrafshan, Seyedeh Nahid Seyedhasani, Fereshteh Najafi, Mostafa Hadei, Jalil Momeni, Ghasem Moradi, Mohammad Sarmadi

**Affiliations:** 1 Department of Environmental Health Engineering, Neyshabur University of Medical Sciences, Neyshabur, Iran; 2 Department of Epidemiology, Johns Hopkins Bloomberg School of Public Health, Baltimore, MD USA; 3 Department of Environmental Health Engineering, School of Health, Torbat Heydariyeh University of Medical Sciences, Torbat Heydariyeh, Iran; 4 Health Sciences Research Center, Torbat Heydariyeh University of Medical Sciences, Torbat Heydariyeh, Iran; 5 Department of Health Information Technology, School of Paramedical Sciences, Torbat Heydariyeh University of Medical Sciences, Torbat Heydariyeh, Iran; 6 Department of Epidemiology and Biostatistics, School of Public Health, Tehran University of Medical Sciences, Tehran, Iran; 7 Department of Environmental Health Engineering, School of Public Health, Tehran University of Medical Science, Tehran, Iran; 8 Student Research Committee, Torbat Heydariyeh University of Medical Sciences, Torbat Heydariyeh, Iran; 9 Neuroscience Research Center, Torbat Heydariyeh University of Medical Sciences, Torbat Heydariyeh, Iran

**Keywords:** Multiple sclerosis, Human development index, Prosperity index, Socioeconomic factors, Ecology study

## Abstract

**Background:**

Socioeconomic factors may be involved in risk of multiple sclerosis (MS), either indirectly or as confounding factors. In this study two comprehensive indicators reflecting socioeconomic differences, including the Human Development Index (HDI) and Prosperity Index (PI), were used to assess the impact of these factors on the worldwide distribution of MS.

**Methods:**

The data for this global ecological study were obtained from three comprehensive databases including the Global Burden of Disease (as the source of MS indices), United Nations Development Programme (source for HDI) and the Legatum Institute Database for PI. MS indices (including prevalence, incidence, mortality, and disability-adjusted life years) were all analyzed in the form of age- and sex-standardized. Correlation and regression analyses were used to investigate the relationship between HDI and PI and their subsets with MS indices.

**Results:**

All MS indices were correlated with HDI and PI. It was also found that developed countries had significantly higher prevalence and incidence rates of MS than developing countries. Education and governance from the PI, and gross national income and expected years of schooling from the HDI were more associated with MS. Education was significantly related to MS indices (*p* < 0.01) in both developed and developing countries.

**Conclusion:**

In general, the difference in income and the socioeconomic development globally have created a landscape for MS that should be studied in more detail in future studies.

**Supplementary Information:**

The online version contains supplementary material available at 10.1186/s12883-021-02170-3.

## Background

Multiple sclerosis (MS) is a chronic inflammatory, demyelinating and neurodegenerative disease of the central nervous system (CNS) that usually starts in the third or fourth decades of life [1–3]. MS has a complex etiology and its causes are currently not fully understood, but it is known that it is one of the leading reasons of non-traumatic neurological disability in young adults, leading to remarkable socioeconomic impacts and the need for lifetime support and management [4–6]. It is estimated that about 2.2 million people are suffering from MS worldwide [7]. There is a broad variation in the prevalence and incidence of MS in different areas of the world [8, 9], supporting the hypothesis that environmental and genetic interaction may play a role in the etiology of MS [10–12]. Some possible risk factors include residential latitude, ultraviolet radiation, intake of vitamin D, Epstein–Barr virus and infectious mononucleosis, and some other non-infectious factors [13].

On the other hand, the rapid economic growth of countries causes changes in lifestyle, hygienic and psychosocial conditions [14]. Researches have demonstrated that countries with better social and economic situations have higher MS prevalence [15–17]. Socioeconomic factors such as education level, life expectancy, and life course socioeconomic position, may be linked to MS incidence and its subsequent progression [18]. Moreover, reported MS incidence is higher in high-income countries [19, 20]. For instance, recently published results from the Global Burden of Disease (GBD) 2016 study showed substantial associations between some neurological disorders, such as MS, and socio-demographic index (SDI) [7], whilst other studies found no significant social gradient or inverse results [21, 22]. In addition, adverse socioeconomic position in childhood has been linked with a proinflammatory phenotype [23], and may be an important factor to consider for complex neuroinflammation and neurological diseases such as MS [23–25]. Therefore, it is of critical importance to comprehend and develop disease-modifying strategies.

The Human Development Index (HDI) and Prosperity Index (PI) are two factors of the socioeconomic situations in countries, and have been previously utilized to study associations between socioeconomic factors and with various diseases, such as diabetes and cancer [26, 27]. However, to the best of our knowledge, these measures have not yet been used in MS studies. HDI is a comprehensive indicator of socioeconomic differences between countries, and PI is an integrated indicator consisted of community-level social well-being based on the state of health services, environmental conditions, and governmental power. Taken together, these two indices represent the extent of countries’ development and, given the importance of these indices in the distribution of other diseases, this study was designed to evaluate their impact on the global distribution of MS.

## Methods

The present study is a global ecological study to analyze the correlation between PI, HDI and their components, and MS prevalence, incidence, mortality, and disability-adjusted life years (DALYs).

### MS data

MS data for all countries in 2017 was acquired from Institute for Health Metrics and Evaluation Global Health Data Exchange (http://ghdx.healthdata.org/). All data analyses were performed with regard to age-standardized rates of MS in for both sexes and each country. The GBD database consists of the data from national and international registries, along with estimates burden of disease for hundreds of health outcomes, and is freely available for researchers [28].

### Prosperity and human development indices

PI is a complex index measuring prosperity of countries not only by one parameter such as economic growth, but also by use of nine components (i.e. business environment, education, economic quality, governance, health, natural environment, personal freedom, safety and security, and social capital. The definitions of each term are listed in The Legatum Prosperity Index™ 2018 [29].

The PI values and rankings data from 149 countries in 2017 were downloaded from the Legatum Institute website (https://www.prosperity.com/). In this report, PI is classified into four categories: low (PI< 50.543), medium (50.543 ≤ PI< 57.570), high (50.570 ≤ HDI < 63.912), and very high (HDI ≥ 63.912) (Supplementary Figure S1 and S2).

HDI scores measuring development of countries were acquired from the United Nations Development Programme (UNDP) database (http://hdr.undp.org/en/data) [30]. HDI ranges from 0 to 1, and components include mean and expected years of schooling, gross national income per capita, and life expectancy at birth (LE) (See the definition of parameters in the supplementary file). In this database, HDI is classified into four categories: low (HDI < 0.556), medium (0.556 ≤ HDI < 0.700), high (0.700 ≤ HDI < 0.800), and very high (HDI ≥ 0.800) [30]. The United Nations considers countries with HDI ≥0.788 as “developed”, and any score below that as “developing” [31]. While HDI has improved in all groups and regions, more rapid increase has been observed in low and medium HDI countries, resulting in less inequitable health systems in certain countries. However, reported national averages may conceal remarkable variations and disparities within countries of both northern and southern hemispheres, as well as increase in income inequality [32–34].

### Statistical analysis

Age-standardized rates of MS indices (incidence, prevalence, mortality, DALY) were stratified by global region. Mean (95% CI) incidence, prevalence, mortality, and DALY of MS was also calculated stratified by HDI categories. Maps of age-standardized incidence rates of MS, PI, and HDI were also created using ArcGIS 10.3 mapping software. We also assessed correlation between MS indices and HDI and PI and their components using Pearson correlation coefficients. In addition, the statistical significance of differences in MS indices among in developing and developed countries was assessed using independent-sample t-tests. On the other hand we used of One-Way ANOVA test to compare the means of more than two groups. We used multivariable linear regression to mutually adjust for HDI and PI components in relation to MS indices.

## Results

**Fig. 1:**
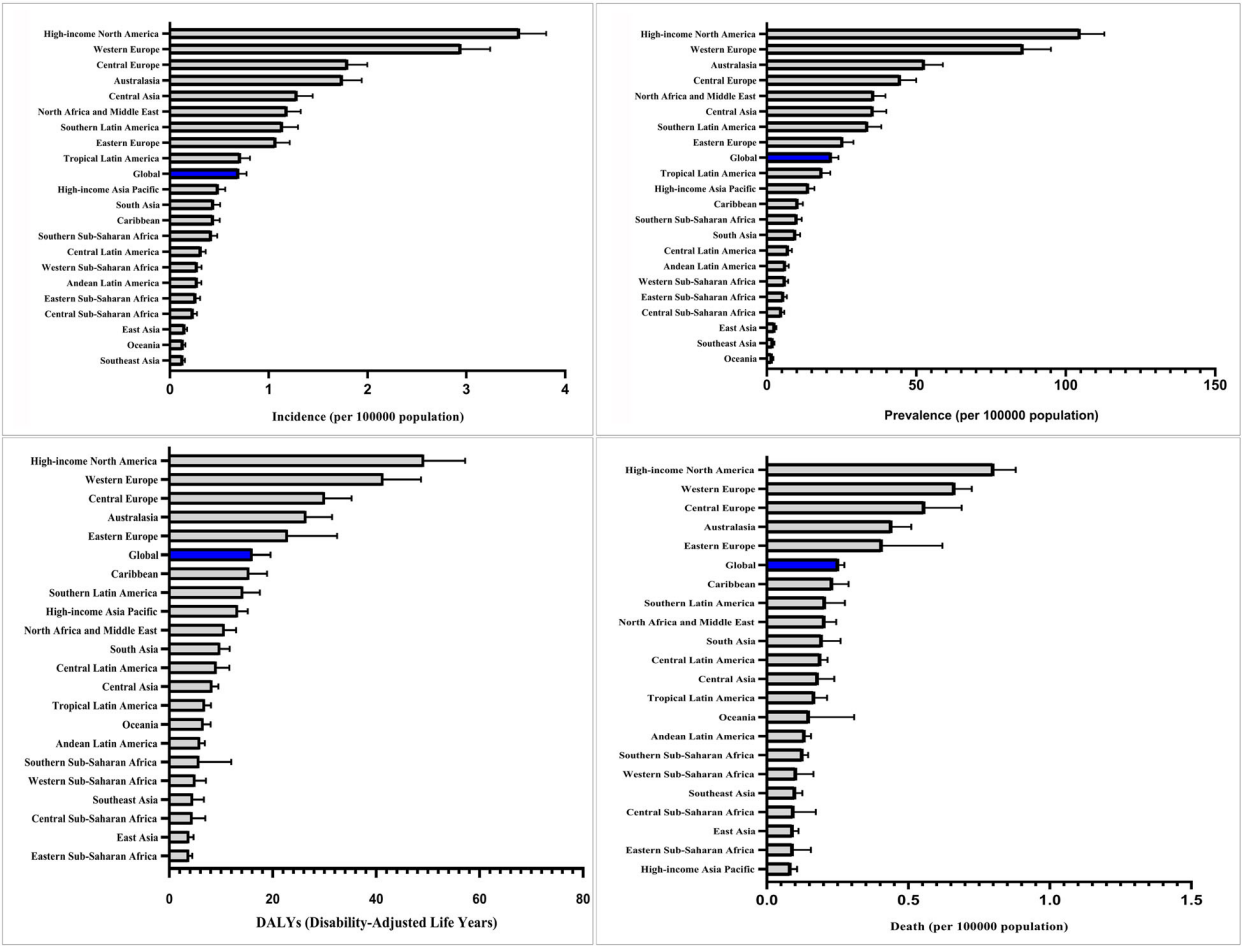
Global and regional Age-standardized of MS indices in 2017 obtained from the Institute for Health Metrics and Evaluation Global Health Data Exchange

Estimates on the frequencies of MS for both sexes were available in GBD for 195 countries. In 2017, an estimated number of 1,761,078 (95% uncertainty interval (UI), 1,598,225–1,947,909) people worldwide had MS. Global MS prevalence was 21.70 (95% UI, 19.69–23.98) per 100,000 persons according to age-standardized rate data (29.34 (95% UI, 26.57–32.43 for female and 13.77 (95% UI, 12.42–15.32). Global MS incidence was 0.70 cases (95% UI, 0.64–0.78) per 100,000 persons (0.90 (95% UI, 0.82–1.00 for female and 0.77 (95% UI, 0.42–0.32). Age-standardized female/male prevalence ratio (F/M) was 2.13. Age-standardized F/M ratio of mortality was 1.32. Global MS mortality was 0.25 cases (95% UI, 0.22–0.27) per 100,000 persons (Fig. 1).

Canada had the highest prevalence (168 cases (95% CI 142.22–197.95) per 100,000) and incidence rates (5.63 cases (95% CI 4.84–6.53) per 100,000). On the other hand, Maldives had the lowest prevalence (1.52 cases (95%CI 1.29–1.80) per 100,000) and incidence (0.09 cases (95% CI 0.08–0.1) per 100,000). UK had the highest age-standardized mortality rate of MS (1.21 (95% CI 0.83–1.31) per 100,000) with a number of 1294 MS mortality cases but USA had highest mortality of with 4019 MS-attributed deaths in 2017.

Among all the countries with available PI, Norway and Yemen had the highest and lowest PIs with 79.85 and 36.36 in 2017, respectively (Supplementary Figure S1, S2). Figure S1 in the supplementary shows PI values with the components in 2017. Also, the highest and lowest HDI were observed in Norway (0.953) and Niger (0.354), respectively (Supplementary Figure S2).

The mean values of MS indices in our study based on HDI categories are presented in Table 1. With the increasing in HDI category, MS indices also has increased. The rate ratio of incidence, prevalence, DALY and mortality in countries with overall high HDI category to those with overall low HDI were 5.38, 6.58, 4.57, and 3.86, respectively.

**Table 1:** Mean (95%CI) MS indices in countries within different HDI categories

MS indices	Low HDI	Medium HDI	High HDI	Very High HDI
**Incidence** ^**a**^	0.36 (0.28–0.43)	0.40 (0.31–0.50)	0.67 (0.50–0.83)	1.92 (1.56–2.28)
**Prevalence** ^**a**^	8.25 (6.13–10.36)	9.52 (6.76–12.28)	17.08 (12.48–21.69)	54.27 (43.44–65.11)
**Mortality** ^**a**^	0.13 (0.11–0.14)	0.14 (0.13–0.16)	0.22 (0.18–0.27)	0.48 (0.41–0.56)
**DALY** ^**a**^	6.34 (5.27–7.42)	7.22 (6.23–8.22)	11.83 (9.40–14.25)	28.96 (24.36–33.56)

^a^ unit of measure is per 100,000 persons-years

**Fig. 2:**
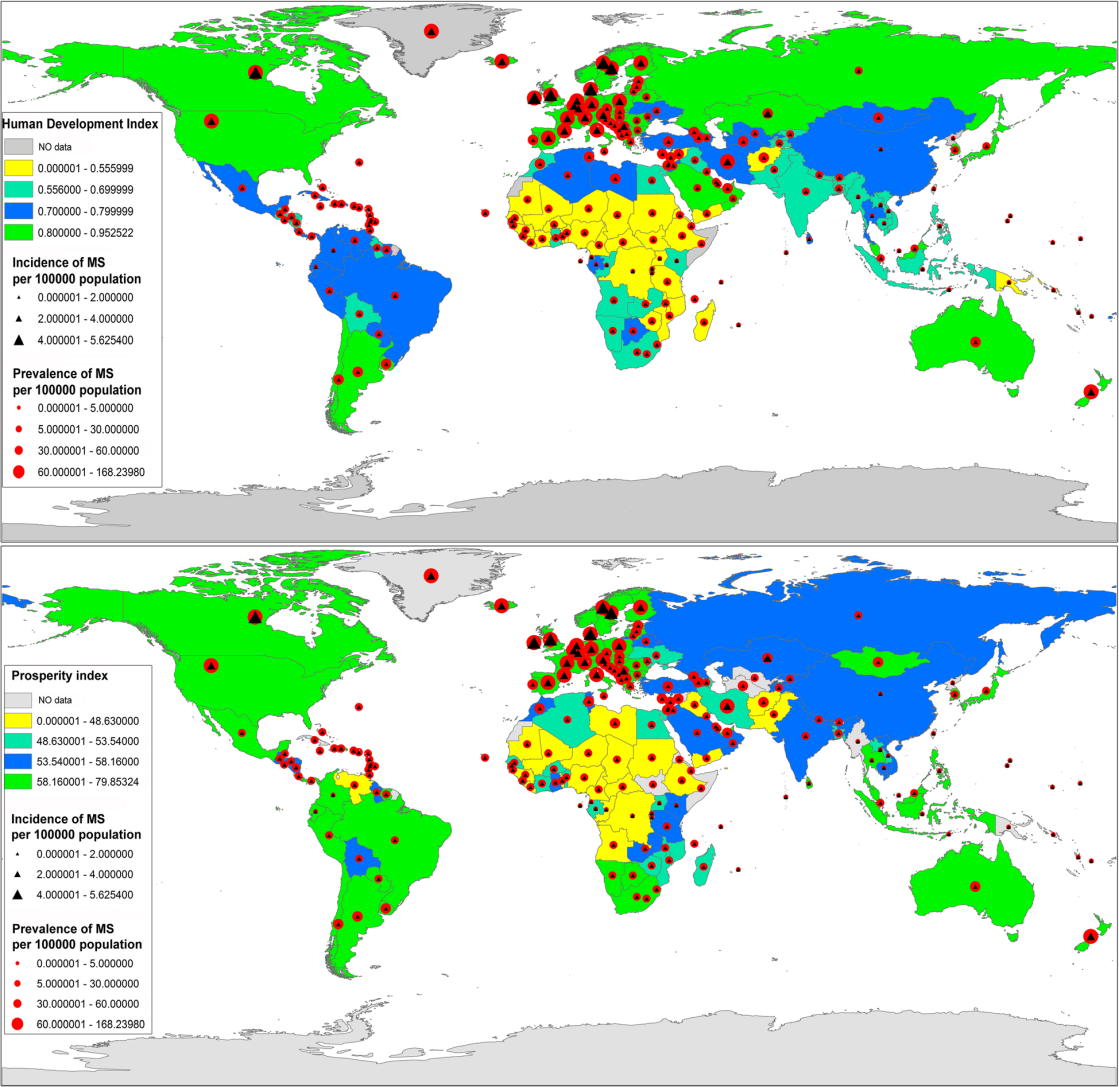
Distribution map of the age-standardized prevalence and incidence of MS, PI and HDI in 2017 obtained from the Institute for Health Metrics and Evaluation Global Health Data Exchange

It can be observed from Figure S2 in the supplementary appendix that more developed countries (with higher overall HDI and PI) are facing higher rates of MS prevalence and incidence; however, in countries with HDI < 0.5 and PI< 50.5, lower rates of prevalence and incidence have been recorded. The PI and HDI distribution GIS map (Fig. 2) illustrates that the countries located in Northern America and Western Europe have the highest prevalence and incidence of MS.

The results of latitude classification in the northern and southern hemispheres showed that countries with higher latitudes have higher MS indices (Table 2). There was also a significant difference between overall PI and HDI in low latitudes (< 20 degrees) and high latitudes (> 40 degrees) in the northern hemisphere (*p* < 0.01).

**Table 2:** Mean (95%CI) MS indices in countries within different latitude categories

Hemisphere	Latitude category	Variables	Mean	95%CI		***P***
**North hemisphere**	**< 20**	**Incidence**	0.34	0.26	0.42	**< 0.001**
		**Prevalence**	7.85	5.88	9.81	
		**DALY**	7.37	5.98	8.76	
		**Mortality**	0.16	0.13	0.18	
		**HDI**	0.62	0.58	0.67	
		**PI**	54.14	51.57	56.72	
	**20–40**	**Incidence**	0.88	0.69	1.06	
		**Prevalence**	23.96	18.36	29.57	
		**DALY**	13.46	11.02	15.90	
		**Mortality**	0.22	0.18	0.26	
		**HDI**	0.69	0.78	0.69	
		**PI**	53.00	58.93	53.00	
	**≥40**	**Incidence**	2.11	1.71	2.50	
		**Prevalence**	58.94	46.81	71.07	
		**DALY**	31.56	26.55	36.56	
		**Mortality**	0.53	0.44	0.61	
		**HDI**	0.82	0.87	0.82	
		**PI**	63.88	69.54	63.88	
**South hemisphere**	**< 20**	**Incidence**	0.26	0.21	0.31	**< 0.01**^**a**^
		**Prevalence**	5.57	4.24	6.90	
		**DALY**	5.61	5.01	6.21	
		**Mortality**	0.13	0.11	0.14	
		**HDI**	0.52	0.64	0.52	
		**PI**	49.16	54.71	49.16	
	**20–40**	**Incidence**	0.81	0.43	1.20	
		**Prevalence**	22.87	10.50	35.25	
		**DALY**	12.26	6.92	17.59	
		**Mortality**	0.20	0.12	0.28	
		**HDI**	0.68	0.85	0.68	
		**PI**	57.55	68.91	57.55	

All of country in south hemisphere was between ranges of 0–40 degree

^a^ not significant for mortality

### Association of MS indices with PI and HDI

The prevalence, incidence, DALY and mortality rates due to MS were positively and significantly correlated with overall PI and HDI (*p* < 0.01), with slightly higher correlation of MS and DALY (Table 3).

**Table 3:** Correlation between PI, HDI and MS indices in 2017

MS indices	Incidence ^a^	Prevalence ^a^	Mortality ^a^	DALY ^a^
HDI	0.62^**^	0.62^**^	0.62^**^	0.65^**^
PI	0.68^**^	0.68^**^	0.66^**^	0.69^**^

*PI* prosperity index, *HDI* human development index

**P < 0.01

^a^ unit of measure is per 100,000 persons-years

In subgroup analysis, the results demonstrated that the MS incidence and prevalence were significantly and positively correlated with all components of PI and HDI (*p* < 0.01) (Table 4).

**Table 4:** Correlation between PI and HDI components and MS variables in 2017

MS variables	Incidence ^**a**^	Prevalence ^**a**^	DALY ^**a**^	Mortality ^**a**^
	r	r	r	r
**Expected Years of Schooling**	0.599**	0.600**	0.629**	0.603**
**Life Expectancy**	0.548**	0.552**	0.564**	0.527**
**Gross National Income**	0.585**	0.595**	0.576**	0.526**
**Mean Years of Schooling**	0.388**	0.386**	0.403**	0.376**
**Economic Quality**	0.593**	0.596**	0.595**	0.563**
**Business Environment**	0.600**	0.605**	0.597**	0.566**
**Governance**	0.665**	0.672**	0.660**	0.628**
**Education**	0.628**	0.625**	0.646**	0.618**
**Health**	0.544**	0.550**	0.538**	0.502**
**Safety and Security**	0.591**	0.586**	0.601**	0.580**
**Personal Freedom**	0.562**	0.564**	0.581**	0.581**
**Social Capital**	0.497**	0.513**	0.464**	0.422**
**Natural Environment**	0.471**	0.473**	0.494**	0.490**

^**^ *P* < 0.01, ^a^: unit of measure is per 100,000 persons-years

Table 5 presents the results of regression analyses of overall HDI and PI on MS incidence, prevalence, DALY, and mortality.

**Table 5:** Regression coefficients for mutually adjusted associations between MS indices and PI and HDI (PI, HDI and latitude adjusted with together)

MS indices	Independent variables	B	***P***	95% CI
**Incidence** ^**a**^	**HDI**	−0.39	0.58	− 1.79, 0.99
	**PI**	0.05	< 0.001	0.03, 0.07
**Prevalence** ^**a**^	**HDI**	−12.45	0.56	−54.69, 29.78
	**PI**	1.45	< 0.001	0.81, 2.10
**DALY**	**HDI**	0.13	0.99	−18.11, 18.37
	**PI**	0.56	< 0.001	0.40, 0.60
**Mortality** ^**a**^	**HDI**	−0.23	0.89	−0.36, 0.31
	**PI**	0.01	< 0.001	0.004, 0.015

^a^: unit of measure is per 100,000 persons-years

The results demonstrated a positive association of overall HDI (adjusted for PI) on DALY (B (SE) = 25.90, *p* = 0.02). There were no statistically significant associations for overall HDI and other variables. In case of PI (adjusted for HDI), significant associations were found for all MS indices (*p* < 0.01). In addition, regression models performed on HDI subgroups showed the positive associations of expected years of schooling and gross national income on all MS indices (Supplementary Table S1). On the other hand, the results for PI subgroups demonstrated that education and governance were positively associated with MS prevalence. Education level was also associated with all MS indices (Supplementary Table S2).

One-way ANOVA tests demonstrated that all MS indices differed significantly among countries in different HDI levels (*p* < 0.01). The results of post hoc tests demonstrated that the difference between the averages of MS indices in the countries with very high HDI was significantly lower than other categories (*p* < 0.01). However, no significant associations were found for high, medium, and low HDI countries (*p* > 0.05) (Supplementary Figure S3).

### MS and countries classification (developed vs. developing)

**Fig. 3:**
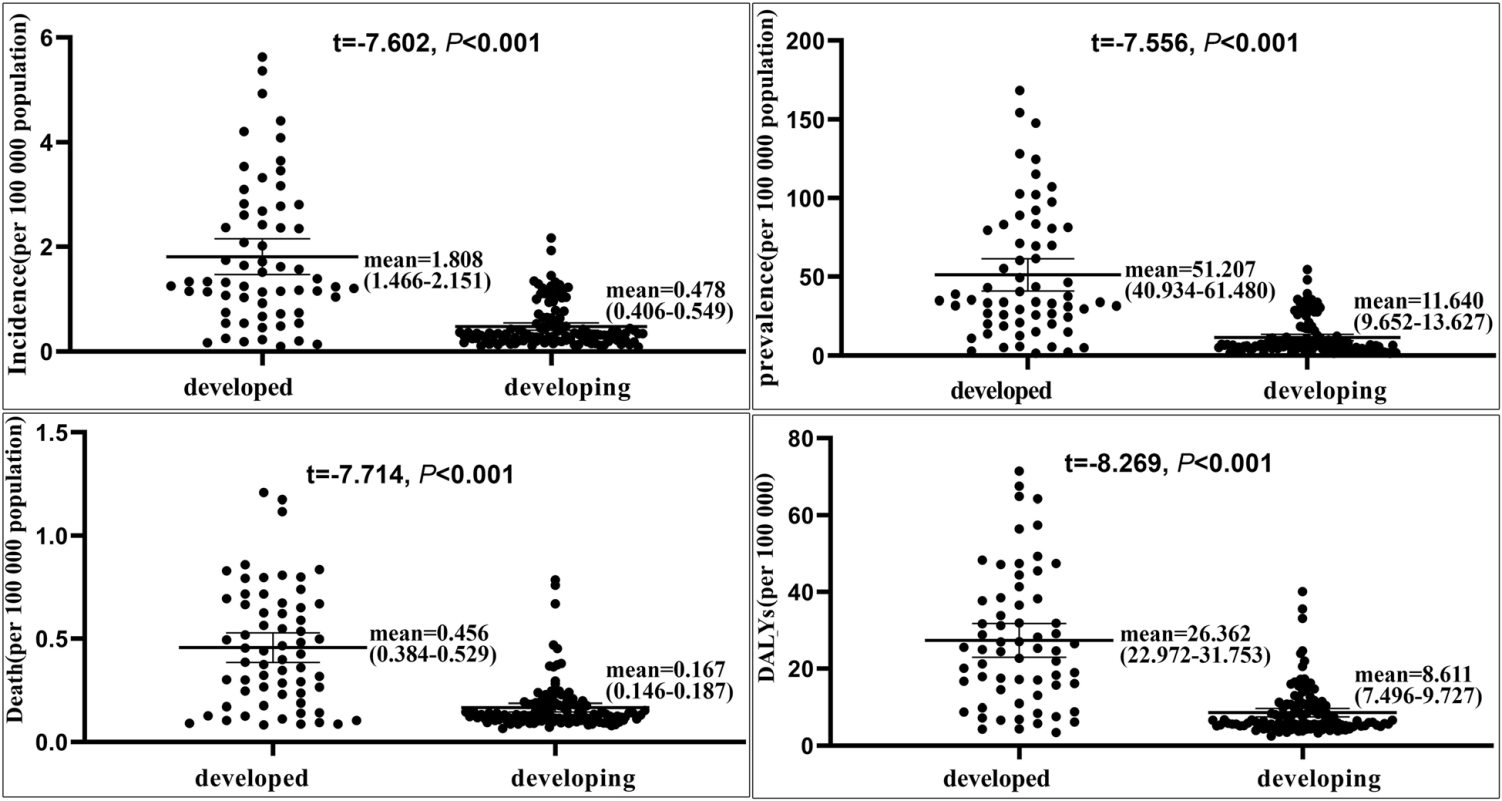
Differences of MS indices in developed and developing countries by independent sample t-test. MS indices in developed countries was significantly higher than those in developing countries. Horizontal lines represent group means with 95% CI

Additional analyses were performed for country categories (developed and developing) in order to investigate the impact of independent variables on MS indices. There were significant differences between MS indices in developed and developing countries (*p* < 0.01) (Fig. 3). Among developing countries, the average of prevalence was 11.64 (CI: 9.65–13.63), which increased with increasing national HDI (*r* = 0.351, *p* < 0.01). Also, MS prevalence in developed countries was 51.21 (CI: 40.93–61.48), and increased with the increase in national HDI (*r* = 0.706, *p* < 0.001).

Our results revealed that the correlations between all MS indices and HDI in developing countries were significant (Supplementary Table S3). Also, regression models demonstrated a significantly positive association with HDI and a negative association with PI for all MS indices (Supplementary Table S4). In developed countries, positive associations of HDI with MS incidence and prevalence were found, but there were no statistically significant relationships between DALY and mortality rate of MS with PI and HDI. In analysis of PI subgroups, education variable showed positive effect on all MS indices in developed and developing countries (Supplementary Table S5 and Table S6).

## Discussion

This study aimed to investigate the association of HDI and PI with MS indices including incidence, prevalence, mortality rate and DALY globally. In the case of MS indices, we found some differences in different parts of world, indicating different patterns of incidence, prevalence and mortality due to MS. The highest rates of MS mortality were observed in developed countries (high-income North American and West European countries). This is possibly because of higher incidence and prevalence in these countries, leading to more deaths and greater DALY. On the other hand, the lowest mortality rates were not observed in areas with low incidence and prevalence. For instance, although the lowest incidence and prevalence are seen in Oceania and Southeast Asia, mortality rates in these areas were higher than those in Asia-Pacific high-income countries. This could be attributed to the differences in healthcare systems in these countries, including quality of care and access to healthcare [35].

Generally, in the last two decades, the prevalence of MS has significantly increased throughout the world [36, 37], along with the incidence and prevalence of disease at the community level [7, 38–40]. It could be related to improvements in the economic level and fast-changing lifestyle of communities. Research has suggested that long-term population growth can increase prevalence and incidence of other chronic diseases [41, 42]. This can vary in different parts of the world due to socioeconomic and cultural factors accompanied with governmental policies for population planning and control.

Figure 2, shows that countries with higher PI and HDI indexes generally have higher prevalence and incidence of MS. In case of other non-communicable diseases such as different types of cancer, higher HDI is related to better accessibility to diagnostic facilities, likewise, higher prevalence of MS could be linked with more updated and available health care facilities in developing countries.

There are also significant differences in prevalence and incidence and subsequent mortality between developed and developing countries, taking into account HDI in different regions and countries. The average prevalence of MS in developed and developing countries were 54.21 and 11.64 per 100,000 population. The average incidence rate in developed countries was about 5.5 times that of developing countries. There are several potential reasons for this observed difference. First, the high incidence of the disease and concurrent quality of healthcare can be one of the main reasons for higher prevalence in developed countries. In more developed areas, factors such as better access to diagnostic facilities and subsequent earlier diagnosis, treatment, and a higher surveillance may be major contributors to high prevalence [43, 44]. Additionally, easy access to better healthcare and diagnostics as well as greater awareness about the disease in more developed areas may increase the number of accurately ascertained MS cases [2]. On the other hand, better socioeconomic development is associated with factors such as obesity, higher smoking rates, more physical activity, etc., which have been suggested as potential risk factors of MS [45]. However, in areas with better socioeconomic status, as evident by higher HDI and PI, there appear to be factors that make people more susceptible to MS [35].

It is important to note, however, that these socioeconomic factors and subsequent access to care can also differ between urban and rural areas within the same country. However, studies have shown higher MS incidence and prevalence subjects from rural vs urban areas of Germany [46, 47], Moldova [48], and Norway [49]. These prior studies suggest that the differences observed are more likely due to country specific resources.

A closer look at the information and reports of GBD indicates that in developing countries, infant and child mortality rates are much higher than those in developed countries. In these areas, people with susceptible immune system may not reach adolescence or adulthood. In contrast, in developed countries, since child mortality and infections are much lower, all people have a higher chance of reaching older ages (i.e. they have higher life expectancy, which is one of the main components of HDI). In other words, people with weaker immune systems are more likely to develop autoimmune diseases such as MS at older ages. This hypothesis, which in fact describes the role of welfare and socioeconomic status driven natural selection, is substantially supported by the hygiene hypothesis of increased likelihood of later life disease [50], but should be further studied.

Regarding the relationship between PI and MS indices, it is worth noting that there is a great deal of similarity between HDI and PI, with PI providing more detailed and comprehensive information, and may be a more suitable measurement in future studies (Table 5). Also, the relationship between PI and MS indicators supports the role of economic stability in access to healthcare and subsequent longevity. Among PI subgroups, factors of health status, state stability, and higher education were more significantly associated with MS indices. These results suggest that countries with greater access to health services, information, education, and subsequent awareness have higher incidence, prevalence, and mortality of MS [35]. Additionally, after controlling for the role of latitude, PI as an index of welfare was the most important statistically effective indicator in the distribution of MS. The role of socioeconomic factors and other concurrent risk factors warrants more detailed studies.

We acknowledge that there are several limitations to the results presented here. As an ecological study, there is the inherent issue of no assumption of temporality. However, as we expect the PI and HDI components vary only slightly from year to year, and rankings of economic stability of countries have remained relatively consistent during the last decade, indices of prosperity and human development may provide more temporally relevant information than expected for many ecological studies. As country-level data, the data used in this study do not account for variability across the latitudinal range in lager countries or differences by urbanity and ethnic distributions in different cities within countries. The data used in this study also do not account for individual characteristics that may influence disease risk or subsequent mortality. Thus, it should be noted that individual genetic, lifestyle, and environmental factors could affect distribution patterns of MS. Nevertheless, studies of individual economic factors may also overlook important group-level determinants of disease. Although the present study provides a promising perspective on MS disease and used incidence data, it should be noted that there may be some measurement error due to differences in how data were collected for different countries and may underestimate or overestimate the actual surveillance reports. Furthermore, data used for this study were previously collected, and we have no information on differences in provision of medical care (i.e. private versus public insurance), which may influence the timing of diagnosis and mortality rate and result in residual confounding.

Although this global analysis provides substantial information on hypothesized factors influencing MS incidence, prevalence, quality of life, and mortality by country, future analyses at the smaller geographic levels – city, province, parish, etc.- could utilize other local data sources and area-based resources. These data sources also do not account for the influence of migration of MS cases to other geographic areas to attain better access to care, which may impact reported MS incidence, prevalence, and mortality rates, changing the risk profile in less developed countries. Future studies should also use data on lifestyle and environmental factors such as air pollution along with other psychosocial stressors, including hostility, violence, food availability, and employment. Despite these limitations, we believe that our use of reliable and validated surveillance data obtained from various government and community sources strengthens the results of this global study and generates several noteworthy findings contributing to the body of knowledge on factors that influence MS epidemiology and surveillance, especially for frequently understudied populations.

## Conclusions

The present study showed that the prevalence of MS is increasing worldwide and developed regions and countries are facing this issue at a higher magnitude. Socioeconomic factors also appear to strongly correlate with the development of the disease, although the exact mechanism is still unclear. It also appears that socioeconomic factors have created a global perspective and a model of MS based on socioeconomic development and it seems that socioeconomic factors have an important role in multiple sclerosis distribution, globally. Many factors can be involved in this regard. This can be the subject of future studies.

## Supplementary Information

**Additional file 1.**

## Data Availability

All data generated or analysed during this study are included publicly available dataset. MS data analyzed in this study are available in the GBD data tool: http://ghdx.healthdata.org/gbd-results-tool. The main source of Prosperity (PI) and human development indices (HDI) data in the world was the Legatum Prosperity Index™ 2017 and UNITED NATIONS DEVELOPMENT PROGRAMME websites: https://prosperitysite.s3-accelerate.amazonaws.com/3515/1187/1128/Legatum_Prosperity_Index_2017.pdf and http://hdr.undp.org/en/composite/HDI.

## Abbreviations

ANOVA: Analysis of variance
CNS: Central nervous system
DALY: Disability-adjusted life year
GBD: Global Burden of Disease
HDI: Human Development Index
LE: Life expectancy at birth
MS: Multiple sclerosis
UNDP: United Nations Development Programme
PI: Prosperity Index
SDI: Socio-demographic index
