# Supplementary material for: Socioeconomic determinants of global distribution of multiple sclerosis: an ecological investigation based on Global Burden of Disease data
Source: BMC Neurol. 2021 Apr 1;21:145. doi: 10.1186/s12883-021-02170-3 (PMC8015140; doi:10.1186/s12883-021-02170-3)
Supplement: Supplementary file 1 — Additional file 1. [file 12883_2021_2170_MOESM1_ESM.pdf]

Supplementary material for

Socioeconomic determinants of global distribution of multiple sclerosis: an ecological investigation based on Global Burden of Disease data

Vahid Kazemi Moghaddam[1#], Aisha S. Dickerson[2#], Edris Bazrafshan[3,4], Seyedeh Nahid Seyedhasani [4, 5], Fereshteh Najafi [6], Mostafa Hadei[7], Jalil Momeni[8], Ghasem Moradi[8], Mohammad Sarmadi [3,4, 9*]

[1] Department of Environmental Health Engineering, Neyshabur University of Medical Sciences, Neyshabur, Iran
[2] Department of Epidemiology, Johns Hopkins Bloomberg School of Public Health, Baltimore, Maryland, USA
[3] Department of Environmental Health Engineering, School of Health, Torbat Heydariyeh University of Medical Sciences, Torbat Heydariyeh, Iran
[4] Health Sciences Research Center, Torbat Heydariyeh University of Medical Sciences, Torbat Heydariyeh, Iran
[5] Department of Health Information Technology, School of Paramedical Sciences, Torbat Heydariyeh University of Medical Sciences, Torbat Heydariyeh, Iran
[6] Department of Epidemiology and Biostatistics, School of Public Health, Tehran University of Medical Sciences, Tehran, Iran
[7] Department of Environmental Health Engineering, School of Public Health, Tehran University of Medical Science, Tehran, Iran
[8] Student Research Committee, Torbat Heydariyeh University of Medical Sciences, Torbat Heydariyeh, Iran
[9] Neuroscience Research Center, Torbat Heydariyeh University of Medical Sciences, Torbat Heydariyeh, Iran

The definition of Human Development Index (HDI) and its components based on information of the United Nations Development Programme (UNDP) database (http://hdr.undp.org/en/data)

HDI is a complex index measuring development of countries not only by economic growth but also by three variables include: having a decent standard of living (income index: gross national income (GNI) per capita), long and healthy life (health index: life expectancy at birth) and being knowledgeable (education index: mean and expected years of schooling).

Method for calculating HDI. The following three indices are used(1):

- Health index: Life expectancy at birth (LEB)
- Education index: Mean and Expected years of schooling (MYS and EYS)
- income index: GNI per capita (PPP US$)

A. Life Expectancy Index (LEI) $=\frac{LE-20}{85-20}$

LEI is 1 when LEB is 85 and 0 when LEB is 20.

B. Education Index (EI) $= \frac{MYSI+EYSI}{2}$

Mean Years of Schooling (MYS) Index $=\frac{MYS}{15}$

15 is the projected maximum of this indicator for 2025.

Expected Years of Schooling (EYSI) Index $= \frac{EYS}{18}$

18 is equivalent to achieving a master's degree in most countries.

C. Income Index (II) $= \frac{\ln(GNIpc)-\ln(100)}{\ln(75000)-\ln(100)}$

II is 1 when GNI per capita is $75,000 and 0 when GNI per capita is $100.

HDI is the geometric mean of the previous three normalized indices:

$$HDI = \sqrt[3]{LEI.EI.II.}$$

LE: Life expectancy at birth
MYS: Mean years of schooling (i.e. years that a person aged 25 or older has spent in formal education)
EYS: Expected years of schooling (i.e. total expected years of schooling for children under 18 years of age)
GNIpc: Gross national income at purchasing power parity per capita

The definition of Prosperity Index components based on information of The Legatum Prosperity Index^{TM} 2018(2)

1. The Economic Quality pillar ranks countries on the openness of their economy, macro-economic indicators, and foundations for growth, economic opportunity and financial sector efficiency.
2. The Business Environment pillar measures a country's entrepreneurial environment, its business infrastructure, barriers to innovation and labour market flexibility.
3. The Governance pillar measures a country's performance in three areas: effective governance, democracy and political participation and rule of law.
4. The Personal Freedom pillar measures national progress towards basic legal rights, individual liberties and social tolerance.
5. The Social Capital pillar measures the strength of personal relationships, social network support, social norms and civic participation in a country.
6. The Safety & Security pillar ranks countries based on national security and personal safety.
7. The Education pillar ranks countries on access to education, quality of education and human capital.
8. The Health pillar measures a country's performance in three areas: basic physical and mental health, health infrastructure and preventative care.
9. The Natural Environment pillar measures a country's performance in three areas: the quality of the natural environment, environmental pressures and preservation efforts.

The highest and lowest values were observed in Norway and Yemen, respectively (Figure 1). The lowest values for economic quality, governance, and education were observed in Yemen, Yemen, and Central African Republic, respectively. The highest values for the same parameters were recorded in Switzerland, Finland, and Switzerland, respectively.

Figure S1 shows PI with nine component value in 2017.

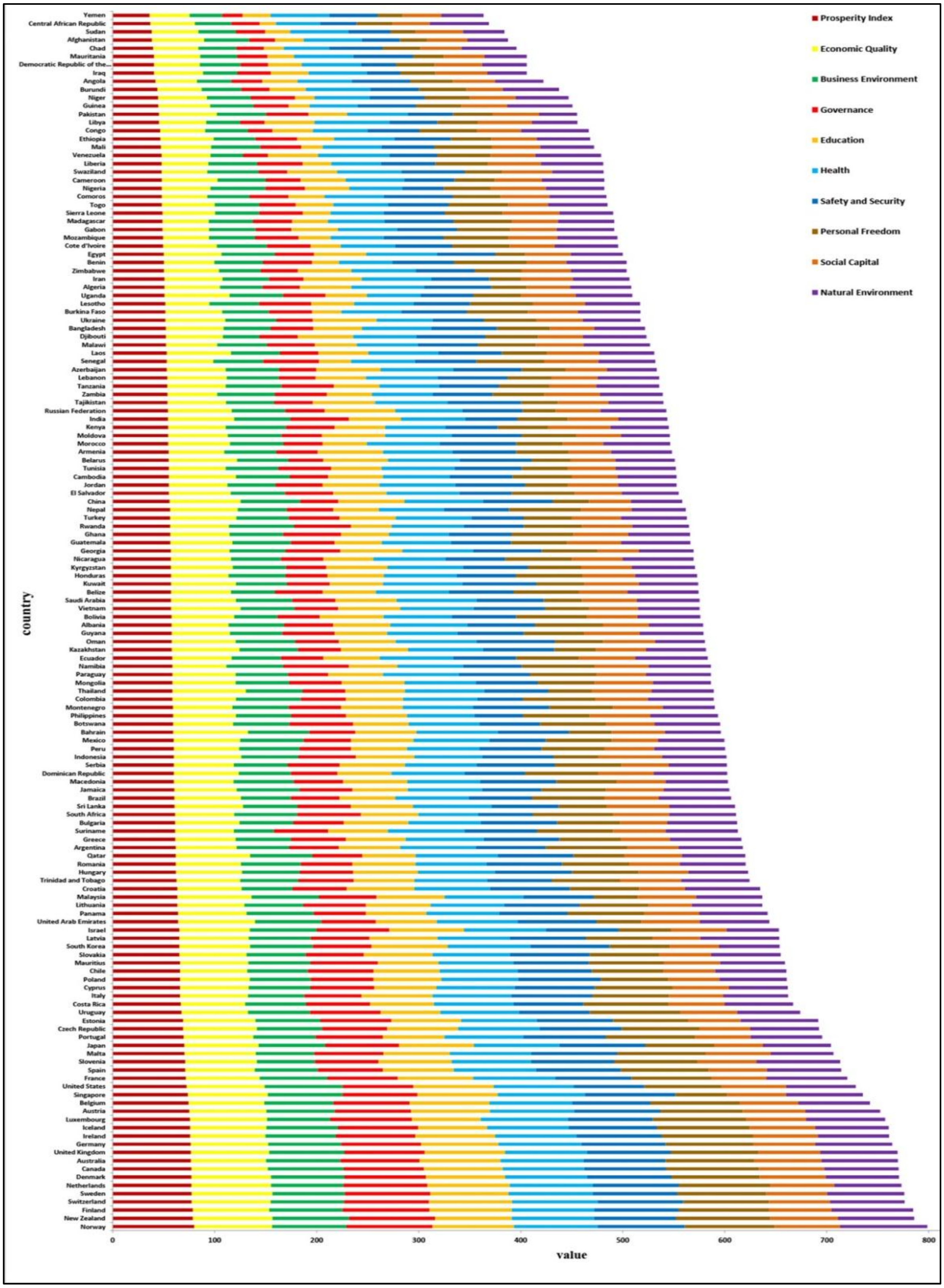

Fig.S1 The value of PI and its subgroups in countries 2017 (3)

ranking	country	Prealence	Incidence	HDI	Prosperity Index	ranking	country	Prealence	Incidence	HDI	Prosperity Index
1	Canada	168.2398	5.8254	0.926	77.03	99	Dominican Republic	9.8017	0.4255	0.7358	60.23
2	Norway	154.441	5.3628	0.9525	79.85	100	Jamaica	9.6347	0.416	0.7322	60.46
3	Sweden	147.7856	4.9284	0.9328	77.59	101	Bangladesh	9.3813	0.4237	0.6082	52.17
4	Ireland	128.2735	4.2098	0.9384	76.12	102	India	9.3782	0.4289	0.6398	54.38
5	Denmark	124.7996	4.4124	0.9295	77.06	103	Mexico	9.2092	0.4021	0.774	59.97
6	United Kingdom	115.1667	4.0891	0.9215	76.92	104	Grenada	9.0275	0.4063	0.7719	
7	Switzerland	107.2866	3.6463	0.944	77.64	105	Belize	8.9741	0.3963	0.7076	57.44
8	Finland	102.7044	3.5383	0.9197	78.46	106	Mauritania	8.8672	0.3867	0.5196	40.58
9	Iceland	102.2164	3.4597	0.9349	76.06	107	Bolivia	8.3693	0.3809	0.6925	57.62
10	United States	97.616	3.3201	0.9239	72.83	108	Namibia	8.3152	0.3621	0.6465	58.64
11	Netherlands	92.1801	3.1659	0.9306	77.33	109	Botswana	8.2678	0.346	0.7166	59.55
12	Luxembourg	89.0937	3.0997	0.9039	75.71	110	Dominica	8.2623	0.3627	0.7151	
13	Germany	83.6122	2.8241	0.936	76.41	111	Saint Lucia	8.1331	0.3549	0.747	
14	France	83.1994	2.681	0.9008	72.01	112	Niger	8.0361	0.3677	0.3539	44.69
15	Belgium	81.3362	2.8051	0.9161	74.24	113	Mali	7.9735	0.3608	0.4269	47.17
16	Austria	80.7853	2.775	0.9078	75.24	114	Madagascar	7.8203	0.3532	0.5192	49.15
17	Greenland	80.2295	2.8025			115	Saint Vincent and the Grenadines	7.6267	0.343	0.7227	
18	Italy	79.5307	2.6086	0.8798	66.2	116	Mozambique	7.57	0.3492	0.4366	49.48
19	New Zealand	71.3263	2.347	0.9167	78.57	117	Cape Verde	7.541	0.3079	0.654	
20	Andorra	69.9273	2.4245	0.8577		118	Eritrea	7.5375	0.3415	0.44	
21	Iran	69.5279	2.3696	0.7981	50.65	119	Zimbabwe	7.5036	0.3294	0.5346	50.37
22	Spain	61.6534	2.0836	0.891	71.42	120	Chad	7.4018	0.344	0.404	39.59
23	Poland	60.3439	2.363	0.8651	66.08	121	Senegal	7.3193	0.3326	0.5051	53.2
24	Kazakhstan	55.2156	2.0168	0.8004	58.14	122	Trinidad and Tobago	7.1722	0.3149	0.7839	62.44
25	Serbia	54.5235	2.1699	0.7867	60.2	123	The Gambia	7.0653	0.3177	0.4601	
26	Australia	49.4708	1.6425	0.9386	76.98	124	Honduras	6.9359	0.3198	0.6167	57.29
27	Albania	48.1335	1.9257	0.7849	57.89	125	Burkina Faso	6.7796	0.3144	0.4234	51.75
28	Turkey	46.4551	1.5675	0.7906	56.28	126	Zambia	6.7729	0.3139	0.5881	53.91
29	Slovenia	43.6533	1.7415	0.8962	71.31	127	Malawi	6.7459	0.3128	0.4766	52.68
30	Hungary	43.0021	1.7132	0.8378	62.3	128	Guinea-Bissau	6.7118	0.3124	0.4553	
31	Czech Republic	40.649	1.6162	0.8876	69.24	129	Guatemala	6.6641	0.3014	0.6503	56.65
32	Turkmenistan	39.212	1.4499	0.7063		130	Angola	6.6143	0.3057	0.5812	42.21
33	Israel	38.9511	1.333	0.9032	65.32	131	Comoros	6.6111	0.2983	0.5033	48.41
34	Slovakia	37.5085	1.5109	0.8552	65.5	132	El Salvador	6.409	0.2821	0.6742	55.47
35	Lebanon	35.7571	1.1944	0.7567	53.57	133	Peru	6.3983	0.2766	0.7498	60.03
36	Armenia	35.5699	1.2729	0.7551	54.83	134	Djibouti	6.3867	0.2943	0.476	52.33
37	Cyprus	35.476	1.2436	0.8688	66.17	135	Nicaragua	6.3177	0.2784	0.6577	56.94
38	Mongolia	35.4258	1.3405	0.7408	58.65	136	Guinea	6.2529	0.292	0.4591	45.06
39	Tunisia	35.066	1.2235	0.7347	55.21	137	Benin	6.0363	0.2783	0.5146	50.37
40	Chile	34.9484	1.1524	0.8429	66.04	138	Nigeria	6.036	0.2644	0.5318	48.2
41	Portugal	34.067	1.2029	0.8471	69.55	139	Costa Rica	5.9564	0.2522	0.7939	66.69
42	Croatia	33.9323	1.3306	0.8311	63.48	140	Guyana	5.8806	0.2677	0.6536	57.91
43	Montenegro	33.8402	1.3886	0.8137	59.04	141	Togo	5.8529	0.2687	0.5032	48.5
44	Afghanistan	33.8004	1.2478	0.4977	38.76	142	Ethiopia	5.8325	0.2748	0.4627	46.8
45	Argentina	33.3518	1.138	0.8248	61.78	143	Suriname	5.8293	0.2602	0.7196	61.29
46	Bosnia and Herzegovina	33.1498	1.3233	0.7685		144	Ghana	5.7521	0.2543	0.5917	56.61
47	Uruguay	33.1003	1.1331	0.8039	67.4	145	Sierra Leone	5.6947	0.2671	0.419	49.08
48	Georgia	32.5584	1.1722	0.7798	56.93	146	Brunei	5.5444	0.2037	0.8533	
49	Latvia	31.7407	1.3169	0.8471	65.35	147	South Sudan	5.5082	0.2524	0.3877	
50	United Arab Emirates	31.579	1.1475	0.8628	64.36	148	Cote d'Ivoire	5.4113	0.2591	0.4923	49.54
51	Syria	31.5715	1.1311	0.5357		149	Tanzania	5.389	0.2516	0.5377	53.59
52	Macedonia	31.2514	1.2876	0.7567	60.31	150	Central African Republic	5.3307	0.2549	0.3668	36.87
53	Estonia	31.0942	1.2476	0.871	69.16	151	Somalia	5.3209	0.2421		
54	Morocco	30.5921	1.099	0.6665	54.65	152	Liberia	5.253	0.2455	0.4351	48.1
55	Libya	30.5023	1.0907	0.7056	45.54	153	Venezuela	5.2434	0.2302	0.7608	47.87
56	Lithuania	29.7334	1.2304	0.8581	63.69	154	Cameroon	5.2365	0.2448	0.5559	48.2
57	Uzbekistan	29.5921	1.102	0.7098		155	Panama	5.2252	0.2262	0.7893	64.19
58	Greece	29.4667	1.0255	0.8699	61.64	156	Singapore	5.0757	0.1851	0.932	73.53
59	Iraq	28.8209	1.031	0.6853	40.6	157	Ecuador	4.8607	0.216	0.7519	58.33
60	Palestine	28.5647	0.9988	0.6858		158	Burundi	4.6531	0.2194	0.4172	43.76
61	Kyrgyzstan	28.4778	1.071	0.6722	57.08	159	Colombia	4.615	0.1991	0.747	58.93
62	Ukraine	27.9565	1.2086	0.7507	51.75	160	Democratic Republic of the Congo	4.6065	0.2191	0.4575	40.39
63	Azerbaijan	27.8619	1.025	0.757	53.33	161	Rwanda	4.5887	0.2112	0.5239	56.5
64	Romania	26.9382	1.1477	0.8112	62.05	162	North Korea	4.531	0.2395		
65	Bulgaria	26.817	1.1674	0.813	61.2	163	Kenya	4.5284	0.2036	0.5899	54.5
66	Algeria	26.2904	0.9417	0.7538	50.82	164	Equatorial Guinea	4.4699	0.1905	0.5906	
67	Belarus	26.0524	1.0697	0.8075	55.09	165	Uganda	4.4039	0.206	0.5163	50.93
68	Malta	25.9094	0.9242	0.8782	70.66	166	Sao Tome and Principe	4.398	0.1933	0.5895	
69	Tajikistan	25.4442	0.9579	0.65	53.99	167	Congo	4.3269	0.2069	0.6063	46.67
70	Russian Federation	24.6485	1.0362	0.8163	54.28	168	Gabon	4.2998	0.1964	0.7022	49.16
71	Egypt	21.288	0.7744	0.6956	49.99	169	Taiwan	4.1545	0.187		
72	Kuwait	21.1232	0.7436	0.8031	57.41	170	Tonga	3.0442	0.1837	0.7256	
73	Bahrain	20.257	0.7488	0.8461	59.61	171	Mauritius	2.9823	0.1694	0.7901	65.9
74	Saudi Arabia	20.221	0.7243	0.8533	57.51	172	Myanmar	2.8216	0.1868	0.5783	
75	Oman	18.9799	0.6724	0.821	58.06	173	China	2.7586	0.1517	0.7517	55.83
76	Moldova	18.6723	0.7879	0.6998	54.61	174	Fiji	2.7393	0.1743	0.7408	
77	Brazil	18.5986	0.7181	0.7592	60.64	175	Northern Mariana Islands	2.6884	0.1495		
78	Jordan	18.0294	0.6527	0.7354	55.28	176	Vietnam	2.6736	0.1535	0.694	57.52
79	Yemen	17.4438	0.6409	0.4519	36.36	177	Vanuatu	2.6384	0.1851	0.6026	
80	Sudan	17.257	0.6379	0.5025	38.39	178	American Samoa	2.5542	0.1513	0.713	
81	Paraguay	15.9208	0.6507	0.7017	58.64	179	Thailand	2.5282	0.142	0.7547	58.91
82	South Korea	15.3186	0.5431	0.9026	65.36	180	Laos	2.5073	0.1689	0.6013	53.09
83	Qatar	15.1455	0.5412	0.8556	62	181	Guam	2.4782	0.1411		
84	Bermuda	14.9676	0.6002			182	Samoa	2.4107	0.15	0.7128	
85	Japan	13.9011	0.4887	0.9092	70.4	183	Seychelles	2.2937	0.137	0.7965	
86	Antigua and Barbuda	12.8369	0.5334	0.7795		184	Cambodia	2.2046	0.1438	0.582	55.27
87	The Bahamas	12.7106	0.5419	0.8071		185	Philippines	2.1873	0.1348	0.6989	59.33
88	Pakistan	12.3657	0.5596	0.5616	45.52	186	Sri Lanka	1.9976	0.1114	0.77	61
89	Cuba	11.774	0.4808	0.7773		187	Marshall Islands	1.9341	0.1387	0.7079	
90	Nepal	11.3647	0.5164	0.574	56.18	188	Solomon Islands	1.9204	0.1342	0.546	
91	Lesotho	11.3162	0.4809	0.5197	51.71	189	Timor-Leste	1.9078	0.1259	0.6249	
92	Puerto Rico	11.1822	0.451			190	Federated States of Micronesia	1.846	0.1279	0.6273	
93	Barbados	11.003	0.458	0.8003		191	Indonesia	1.7859	0.1164	0.694	60.18
94	South Africa	10.8108	0.4467	0.699	61.11	192	Papua New Guinea	1.6993	0.1304	0.5443	
95	Bhutan	10.672	0.4808	0.6124		193	Malaysia	1.6601	0.0968	0.8018	63.69
96	Virgin Islands, U.S.	10.1587	0.4233			194	Kiribati	1.5666	0.1089	0.6118	
97	Haiti	10.1384	0.4685	0.4979		195	Maldives	1.5238	0.0898	0.7169	
98	Swaziland	9.9847	0.4404	0.588	48.13						

No data

incidence≥4 =high
2≤incidence<4 =medium
incidence <2 =low

prevalence ≥30 =high
5≤ prevalence <30 =medium
prevalence <5 =low

HDI ≥ 0.80 =very high
0.70≤HDI<0.80 =high
0.556≤HDI<0.70 =medium
HDI<0.556 =low

PI ≥63.912 =very high
57.570≤PI <63.912 =high
50.543≤PI <57.570 =medium
PI <50.543 =low

Fig. S2 The MS prevalence and incidence, PI and HDI in 195 countries in our study

Table S1 Unstandardized regression coefficients for multivariable associations between MS indices and HDI subgroups in 2017

Variables	Incidence [a]	Prevalence [a]	DALY [a]	Mortality [a]
	B (SE)	B (SE)	B (SE)	B (SE)
**Expected Years of Schooling**	0.12 (0.03)**	3.59 (0.81)**	1.94 (0.37)**	0.03 (0.01)**
**Mean Years of Schooling**	0.04 (0.02)	1.16 (0.65)	0.59 (0.29)*	0.01 (0.01)
**Gross National Income**	$1.84x10^{-5}$ (<0.01)**	0.001 (<0.01)**	<0.01 (<0.01)**	$2.82x10^{-6}$ (<0.01)**

** *P*< 0.01; * *P*<0.05; , a: unit of measure is per 100,000 persons-years

Multivariable regression showed that there are associations between PI subgroups and MS variables. Governance and Education had significant associations with MS variables. Incidence and prevalence were affected mostly by Governance; while, mortality and DALY were influenced mostly by Education.

Table S2 Unstandardized regression coefficients for multivariable associations between MS indices and PI subgroups in 2017

variables	Incidence [a]		Prevalence [a]		DALY [a]		Mortality [a]	
	B (SE)	*P*	B (SE)	*P*	B (SE)	*P*	B (SE)	*P*
**Economic Quality**	<0.001 (0.018)	0.98	-0.041 (0.542)	0.94	0.032 (0.244)	0.90	0.001 (0.004)	0.86
**Business Environment**	-0.007 (0.016)	0.69	-0.194 (0.489)	0.69	-0.094 (0.220)	0.67	-0.001 (0.004)	0.79
**Governance**	**0.030 (0.013)**	**0.02**	**0.920 (0.381)**	**0.02**	**0.343 (0.171)**	**<0.05**	0.004 (0.003)	0.15
**Education**	**0.029 (0.010)**	**0.01**	**0.788 (0.311)**	**0.01**	**0.495 (0.140)**	**<0.01**	**0.008 (0.002)**	**<0.01**
**Health**	-0.016 (0.016)	0.32	-0.336 (0.481)	0.49	-0.346 (0.216)	0.11	-0.007 (0.004)	0.06
**Safety and Security**	0.013 (0.010)	0.21	0.335 (0.309)	0.28	0.190 (0.140)	0.18	0.003 (0.002)	0.16
**Personal Freedom**	0.006 (0.008)	0.46	0.149 (0.232)	0.52	0.134 (0.104)	0.20	0.003 (0.002)	0.06
**Social Capital**	0.004 (0.015)	0.80	0.227 (0.460)	0.62	-0.096 (0.207)	0.64	-0.003 (0.003)	0.39
**Natural Environment**	-0.013 (0.012)	0.29	-0.348 (0.350)	0.32	-0.131 (0.158)	0.41	-0.002 (0.003)	0.51

a: unit of measure is per 100,000 persons-years

MS and countries classification (developed vs developing)

Pearson correlation analysis showed that the subgroups of HDI and PI were significantly associated with MS variables in both developed and developing countries. The relationship in developed countries was stronger.

Table S3 Correlation (r) between MS variables and HDI, PI in Developing and Developed countries

category	Variables	Prevalence [a]	Incidence [a]	DALY [a]	Mortality [a]
**Developing**	**HDI**	0.351**	0.346**	0.400**	0.382**
	**PI**	0.052	0.067	0.091	0.130
**Developed**	**HDI**	0.706**	0.694**	0.673**	0.605**
	**PI**	0.675**	0.664**	0.662**	0.627**
HDI= Human Development Index, PI= Prosperity Index, ** P<0.01, * P<0.05, ; a: unit of measure is per 100,000 persons-years					

Multivariable regression between HDI and PI and MS variables showed that controlling for PI, there is an association between HDI and incidence in both developed and developing countries. Associations with HDI were stronger than PI in both groups of countries. PI in developing countries was inversely associated with MS variables. In developed countries, HDI only significant for incidence and prevalence of MS.

Table S4 Unstandardized regression coefficients for multivariable associations between MS indices and HDI and PI in 2017

Dependent variables	Independent variables	Developing	Developed
		B (SE)	B (SE)
**Incidence [a]**	**HDI**	2.38 (0.44)**	11.11 (5.10)**
	**PI**	-0.03 (0.01)**	0.06 (0.04)
**Prevalence [a]**	**HDI**	69.11 (11.95)**	341.53 (150.59)*
	**PI**	-0.82 (0.22)**	1.72 (1.02)
**DALY [a]**	**HDI**	38.00 (7.23)**	130.65 (66.06)
	**PI**	-0.41 (0.14)**	0.80 (0.45)
**Mortality a**	**HDI**	0.58 (0.14)**	1.60 (1.17)
	**PI**	-0.01 (0.00)**	0.02 (0.01)
** *P*<0.01, * *P*<0.05. HDI= Human Development Index, PI= Prosperity Index, ; a: unit of measure is per 100,000 persons-years			

Multiple regressions showed that there are significant positive relationships between the PI subgroups in developed and developing countries and MS variables.

Table S5 Unstandardized regression coefficients for multivariable associations between MS indices and PI subgroups in developed and developing countries

	Covariates	Incidence [a]	Prevalence [a]	DALY [a]	Mortality [a]
		B (SE)	B (SE)	B (SE)	B (SE)
**Developing**	**Economic Quality**	-0.015 (0.009)	-0.440 (0.235)	-0.176 (0.144)	-0.003 (0.003)
	**Business Environment**	0.003 (0.008)	0.068 (0.222)	0.068 (0.136)	0.002 (0.003)
	**Governance**	0.002 (0.007)	0.100 (0.201)	-0.058 (0.124)	-0.002 (0.002)
	**Education**	**0.014 (0.005)***	**0.355 (0.151)***	**0.270 (0.093)****	**0.004 (0.002)***
	**Health**	0.008 (0.008)	0.294 (0.220)	0.024 (0.135)	<0.001 (0.003)
	**Safety and Security**	0.008 (0.005)	0.185 (0.149)	0.076 (0.092)	0.001 (0.002)
	**Personal Freedom**	0.001 (0.005)	-0.034 (0.132)	0.091 (0.081)	**0.003 (0.002)***
	**Social Capital**	-0.016 (0.008)	-0.447 (0.228)	-0.229 (0.139)	-0.004 (0.003)
	**Natural Environment**	**-0.020 (0.007)****	**-0.488 (0.190)***	**-0.316 (0.117)****	**-0.006 (0.002)****
**Developed**	**Economic Quality**	0.068 (0.060)	1.779 (1.779)	0.932 (0.756)	0.016 (0.013)
	**Business Environment**	-0.067 (0.047)	-1.888 (1.398)	-0.854 (0.594)	-0.012 (0.010)
	**Governance**	0.010 (0.032)	0.269 (0.949)	0.221 (0.403)	0.003 (0.007)
	**Education**	**0.082 (0.028)****	**2.439 (0.846)****	**0.964 (0.360)****	**0.014 (0.006)***
	**Health**	-0.081 (0.055)	-1.823 (1.623)	**-1.711 (0.690)***	**-0.035 (0.012)****
	**Safety and Security**	-0.012 (0.028)	-0.593 (0.837)	0.106 (0.356)	0.007 (0.006)
	**Personal Freedom**	0.014 (0.017)	0.451 (0.505)	0.187 (0.215)	0.003 (0.004)
	**Social Capital**	**0.087 (0.038)***	**2.737 (1.130)***	0.871 (0.480)	0.010 (0.008)
	**Natural Environment**	0.004 (0.024)	0.068 (0.706)	0.188 (0.300)	0.004 (0.005)

** *P*<0.01, * *P*<0.05, ; a: unit of measure is per 100,000 persons-years

Regression model in developed countries showed positive associations of EYS on all MS variables. Additionally, incidence and prevalence of MS were positively associated with LE. The other variables was not statistical significant with HDI subgroups in developed country and all variables in developing country.

Table S6 Unstandardized regression coefficients for multivariable associations between MS indices and HDI subgroups in developed and developing countries

MS variables	covariate	Developing		Developed	
		B (SE)	*P*	B (SE)	*P*
Incidence [a]	Expected Years of Schooling	0.013 (0.022)	0.55	**0.293 (0.082)**	**<0.01**
	Mean Years of Schooling	0.013 (0.013)	0.33	0.030 (0.057)	0.60
	Life Expectancy	0.011 (0.007)	0.11	**0.072 (0.034)**	**0.04**
	Gross National Income	5.343×10^{-6} (<0.001)	0.53	1.131×10^{-5} (<0.001)	0.14
Prevalence [a]	Expected Years of Schooling	0.300 (0.608)	0.62	**8.929 (2.407)**	**<0.01**
	Mean Years of Schooling	0.335 (0.355)	0.35	0.714 (1.675)	0.67
	Life Expectancy	0.335 (0.196)	0.09	**2.321 (0.985)**	**0.02**
	Gross National Income	<0.001 (<0.001)	0.46	<0.001(<0.001)	0.10
DALY	Expected Years of Schooling	0.391 (0.340)	0.25	**3.789 (1.090)**	**<0.01**
	Mean Years of Schooling	0.222 (0.199)	0.27	0.544 (0.758)	0.48
	Life Expectancy	0.181 (0.110)	0.10	0.760 (0.446)	0.09
	Gross National Income	8.678×10^{-5} (<0.001)	0.51	9.091×10^{-5} (<0.001)	0.38
Mortality [a]	Expected Years of Schooling	0.009 (0.006)	0.17	**0.057 (0.019)**	**<0.01**
	Mean Years of Schooling	0.004 (0.004)	0.32	0.008 (0.013)	0.54
	Life Expectancy	0.003 (0.002)	0.15	0.008 (0.008)	0.28
	Gross National Income	1.293×10^{-6} (<0.001)	0.60	8.445×10^{-7} (<0.001)	0.64
a: unit of measure is per 100,000 persons-years					

MS and HDI classification

Based on the data of United Nations Development Programme (UNDP) and the Legatum Institute's data of MS, 186 (out of 195) countries were placed into four groups, including low (N = 39), medium (N = 39), high (N = 51), and very high HDI countries (N = 57). One-way ANOVA test demonstrated that all MS variables differed significantly among countries in different HDI levels (p<0.01). The results of post hoc tests demonstrated that difference between group means of MS variables in the countries with very high HDI was significantly lower than other categories (p < 0.01) (Figure 3), but there was no significant difference between high, medium and low HDI categories (with Graph pad prism software).

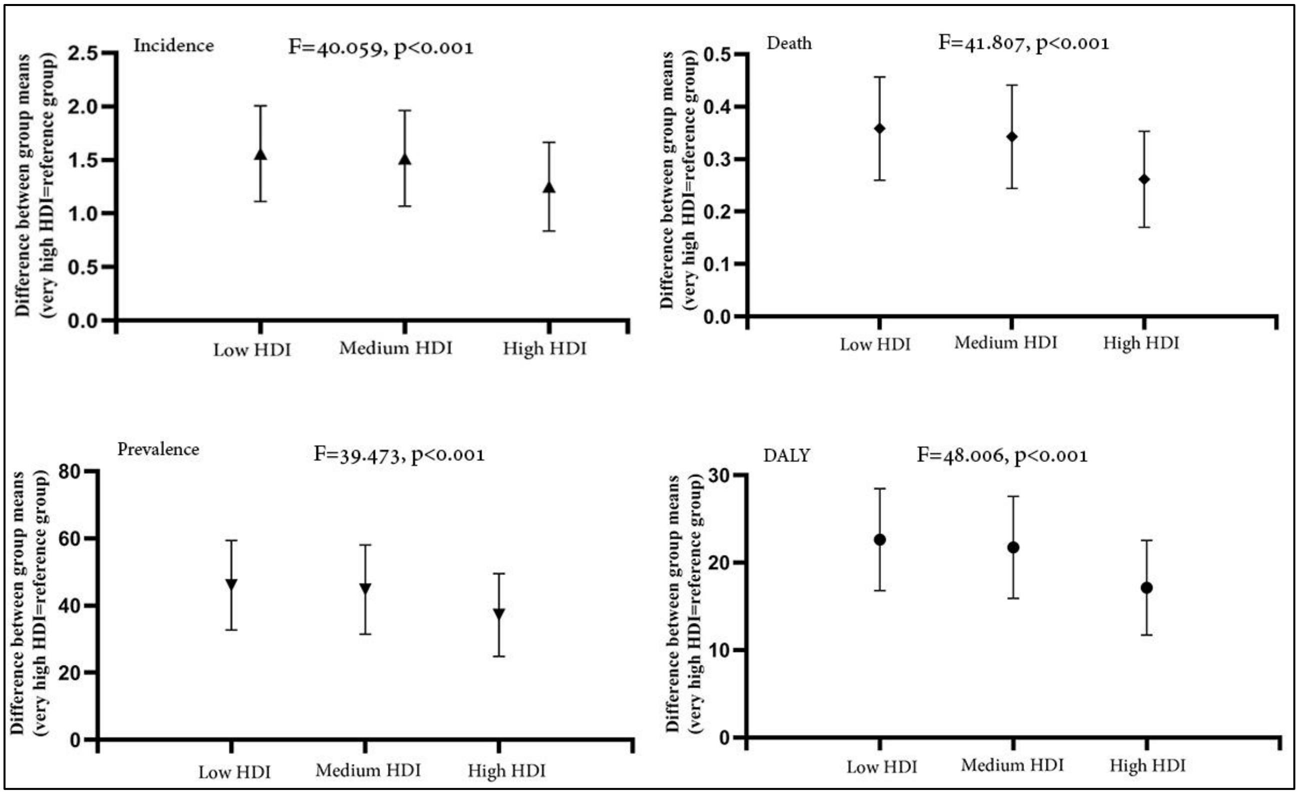

Fig. S3 Differences between group means of MS variables in four HDI groups with ANOVA tests. Differences between group means of MS variables in low, medium and high HDI countries is significantly higher than that of very high HDI countries. Vertical lines represent differences between group means.

References

1. investopedia.com. Human Development Index (HDI) 2019 [cited 2019]. Available from: https://www.investopedia.com/terms/h/human-development-index-hdi.asp.
2. Stroud BP. The Legatum Prosperity Index™ 2018: Legatum Institute; 2019. 76]. Available from: https://prosperitysite.s3-accelerate.amazonaws.com/2515/4321/8072/2018_Prosperity_Index.pdf.
3. Institute L. The Legatum Prosperity Index™ 2019 [cited 2019]. Available from: https://prosperitysite.s3-accelerate.amazonaws.com/3515/1187/1128/Legatum_Prosperity_Index_2017.pdf.
